# Real‐world use of carfilzomib combined with lenalidomide and dexamethasone in patients with multiple myeloma in Europe and Israel

**DOI:** 10.1002/jha2.595

**Published:** 2022-11-06

**Authors:** Xavier Leleu, Eirini Katodritou, Thomas Kuehr, Evangelos Terpos, Jo Caers, Renato Zambello, Alessandra Brescianini, Tony Liang, Sally Wetten, Sorina N. Badelita

**Affiliations:** ^1^ Department of Haematology University Hospital Centre La Miletrie and Inserm Poitiers France; ^2^ Department of Haematology Theagenio Cancer Hospital Thessaloniki Greece; ^3^ Department of Internal Medicine IV Academic Teaching Hospital Wels‐Grieskirchen Wels Austria; ^4^ Department of Clinical Therapeutics, School of Medicine National and Kapodistrian University of Athens Athens Greece; ^5^ Department of Haematology Liège University Hospital Centre Liège Belgium; ^6^ Department of Medicine Haematology and Clinical Immunology Branch, University of Padua Padua Italy; ^7^ Research and Development Department Amgen (Europe) GmbH Rotkreuz Switzerland; ^8^ Department of Biostatistics Parexel International Taipei Taiwan; ^9^ Center for Observational Research Amgen Ltd Uxbridge UK; ^10^ Department of Hematology Fundeni Clinical Institute Bucharest Romania

**Keywords:** carfilzomib, multiple myeloma, proteasome inhibitor, real world, relapsed/refractory

## Abstract

Clinical trials have demonstrated the efficacy and safety of carfilzomib in patients with relapsed/refractory multiple myeloma (RRMM); however, prospective real‐world data are limited. This real‐world, prospective, observational study evaluated carfilzomib use, effectiveness and safety in adults with RRMM. Data are presented for a subset of patients (*n* = 383) who received carfilzomib in combination with lenalidomide and dexamethasone (KRd). The overall response rate (ORR) was 83.6% among 360 evaluable patients. Treatment responses were better when KRd was administered at earlier therapy lines than at later lines of therapy (ORR: second line, 85.3%; third line or later, 81.0%). In patients with the anti‐CD38 antibody‐refractory disease, ORR was higher when KRd was administered earlier than at later therapy lines (second line/third line, 75.0%; fourth line or later, 60.0%). An ORR of 68.1% and 82.0% was achieved in the lenalidomide‐refractory and not lenalidomide‐refractory subgroups, respectively. KRd was consistently administered per the European label (twice weekly dose of 27 mg/m^2^) and the median time to discontinuation was 14.6 months. The safety profile of KRd was consistent with previous studies. These real‐world data highlight the effectiveness of KRd as a treatment for patients with RRMM, including those with disease refractory to lenalidomide or anti‐CD38 antibodies.

## 1 INTRODUCTION

Over the past 15 years, the introduction of multi‐agent treatment regimens that combine immunomodulatory drugs (IMiDs), proteasome inhibitors (PIs), or CD38‐targeting antibodies have substantially improved outcomes for patients with multiple myeloma (MM) [1, 2, 3, 4, 5, 6, 7, 8]. The use of multi‐drug regimens as first‐ or second‐line (2L) therapy is now standard practice;[9] however, this has led to an increasing number of patients with disease that is refractory to multiple classes of agents [7, 10–12]. There is a need to optimise the sequencing of combination therapies across multiple treatment lines, particularly for patients with relapsed/refractory MM (RRMM) for whom effective therapeutic options may be limited [7, 8, 10, 12].

Carfilzomib is a second‐generation PI [13]. In the randomised, phase 3 ASPIRE trial, carfilzomib in combination with lenalidomide and dexamethasone (KRd) improved survival in patients with RRMM compared with lenalidomide and dexamethasone (Rd) (24‐month overall survival: 73.3% vs. 65.0%, respectively; median progression‐free survival: 26.3 months vs. 17.6 months, respectively) [14]. The combination also showed a favourable risk‐benefit profile [14]. Based on these data, KRd was approved for use in 2015 by the European Medicines Agency in patients with MM who had received at least one previous line of treatment [15]. The indication has since expanded to include the use of carfilzomib in combination with dexamethasone alone and in combination with daratumumab and dexamethasone [15].

Although phase 3 randomised controlled trials remain the gold standard for obtaining regulatory approval, they are typically conducted over a short time period (1–2 years), resulting in a lack of information on the long‐term efficacy and safety of a therapy [16]. Furthermore, approximately 40% of individuals with MM in the real world do not meet clinical trial inclusion criteria [16]. Owing to the rapidly changing treatment landscape for MM, treatments evaluated in older clinical trials may be less reflective of recent clinical practice, which includes increased use of daratumumab and lenalidomide [15, 17]. Understanding how treatments are used in the real world provides a valuable additional perspective on the profile of available therapies [16]. Currently, however, data are limited on the use of carfilzomib‐based regimens in European clinical practice.

This study aimed to provide contemporary real‐world data on the use of carfilzomib in adults with RRMM. Here, we report the use, effectiveness and safety in patients with RRMM prospectively treated with KRd in Europe and Israel.

## 2 METHODS

### 2.1 Study design

This real‐world, prospective, observational cohort study (ClinicalTrials.gov identifier: NCT03091127) was conducted at 112 centres in 11 countries (Austria, Belgium, Bulgaria, the Czech Republic, France, Greece, Italy, the Netherlands, Norway, Romania and Israel) between March 2017 and March 2020. Adults (≥18 years of age) who had been prescribed carfilzomib as a 2L or later treatment for MM in routine clinical practice were eligible for inclusion. Patients prescribed carfilzomib as part of a clinical trial or within a compassionate use programme were excluded.

Data were collected from the first administration of carfilzomib until 30 days after the patients’ final dose or 18 months after treatment initiation, death, loss to follow‐up, withdrawal of consent or end of study (31 March 2020), whichever occurred earliest. Baseline data and initial follow‐up time on carfilzomib treatment were collected retrospectively upon enrolment; prospective data, including treatment‐related treatment‐emergent adverse events (TEAEs) of grade 3 and above, were collected at quarterly intervals thereafter until the end of the study.

### 2.2 Outcomes

Study outcome measures included patient demographics and disease characteristics, concomitant medications, response to carfilzomib treatment as assessed by the investigator and according to International Myeloma Working Group (IMWG) criteria [18], carfilzomib use and treatment characteristics, and the safety profile of carfilzomib. Additional outcomes included KRd use described by line of therapy and KRd use in the following subgroups: patients with disease refractory to anti‐CD38 antibodies, and patients exposed to lenalidomide, in any previous line of therapy.

### 2.3 Statistical analysis

Descriptive statistics were used to summarise the data. Two‐sided 95% confidence intervals (CIs) were calculated using Wilson's method, when appropriate. Time to treatment discontinuation and follow‐up time on treatment was estimated using Kaplan–Meier and reverse Kaplan–Meier methodology, respectively. Relative dose intensity was calculated relative to the dosing regimen on the carfilzomib label. Refractory disease was defined as per the International Myeloma Workshop Consensus Panel 1 criteria [19].

## 3 RESULTS

### 3.1 Patient and disease characteristics

In total, 701 patients were enrolled across 11 participating countries in Europe and Israel; of these, 383 patients (54.6%) received KRd and were included in this analysis (Table 1). Most patients (*n* = 230; 60.1%) received KRd at 2L; the remaining patients (*n* = 153; 39.9%) received KRd at third line or later (3L+). The median age at initiation of KRd treatment was 65.0 years. Of the patients with available data, approximately one‐third (30.7%) were considered frail. At diagnosis, cytogenic risk was considered to be high in 14.6% of patients. Patient and disease characteristics were broadly similar for those who received KRd at 2L or 3L+.

**TABLE 1:** Patient demographics and disease characteristics and treatment history at baseline presented overall and by line of therapy

**Characteristic**	**2L (*n* = 230)**	**3L+ (*n* = 153)**	**Overall (*N* = 383)**
Patient and disease characteristics			
Sex, male	133 (57.8)	103 (67.3)	236 (61.6)
Age at carfilzomib initiation, median (Q1– Q3)	65 (56–69)	64 (58–70)	65 (57–70)
ISS stage^a^ at carfilzomib initiation	68 (29.6)	48 (31.4)	116 (30.3)
I^b^	35 (51.5)	22 (45.8)	57 (49.1)
II^b^	18 (26.5)	14 (29.2)	32 (27.6)
III^b^	15 (22.1)	12 (25.0)	27 (23.3)
ECOG PS at carfilzomib initiation	142 (61.7)	89 (58.2)	231 (60.3)
0–1^b^	124 (87.3)	72 (80.9)	196 (84.8)
2–3^b^	18 (12.7)	16 (18.0)	34 (14.7)
4^b^	0 (0.0)	1 (1.1)	1 (0.4)
Derived frailty score^c^	142 (61.7)	89 (58.2)	231 (60.3)
Fit^b^	48 (33.8)	29 (32.6)	77 (33.3)
Intermediate^b^	54 (38.0)	29 (32.6)	83 (35.9)
Frail^b^	40 (28.2)	31 (34.8)	71 (30.7)
Cytogenetic risk at diagnosis	230 (100.0)	153 (100.0)	383 (100.0)
High	41 (17.8)	15 (9.8)	56 (14.6)
Standard/intermediate	42 (18.3)	31 (20.3)	73 (19.1)
Not available	147 (63.9)	107 (69.9)	254 (66.3)
Treatment history			
Number of prior lines of therapy, median (Q1–Q3)	1.0 (1.0–1.0)	2.0 (2.0–3.0)	1.0 (1.0–2.0)
Previous HSCT	142 (61.7)	102 (66.7)	244 (63.7)
Type of previous therapy^d^			
PI	221 (96.1)	151 (98.7)	372 (97.1)
Bortezomib	217 (94.3)	149 (97.4)	366 (95.6)
Ixazomib	3 (1.3)	10 (6.5)	13 (3.4)
Carfilzomib	1 (0.4)	3 (2.0)	4 (1.0)
IMiD	115 (50.0)	139 (90.8)	254 (66.3)
Thalidomide	95 (41.3)	83 (54.2)	178 (46.5)
Lenalidomide	25 (10.9)	106 (69.3)	131 (34.2)
Pomalidomide	1 (0.4)	29 (19.0)	30 (7.8)
Monoclonal antibody	10 (4.3)	25 (16.3)	35 (9.1)
Daratumumab	9 (3.9)	25 (16.3)	34 (8.9)
Isatuximab	1 (0.4)	1 (0.7)	2 (0.5)
Elotuzumab	0 (0.0)	0 (0.0)	0 (0.0)
Refractory to any previous treatment line^e^, ^f^			
Bortezomib	49 (22.6)	63 (42.3)	112 (30.6)
Lenalidomide	8 (32.0)	67 (63.2)	75 (57.3)
Daratumumab	8 (88.9)	24 (96.0)	32 (94.1)
Isatuximab	1 (100.0)	1 (100.0)	2 (100.0)

Data are presented as *n* (%) unless stated otherwise.

Percentages are subject to rounding.

Abbreviations: 2L, second line; 3L+, third line or later; ECOG PS, Eastern Cooperative Oncology Group performance status; HSCT, haematopoietic stem cell transplantation; IMiD, immunomodulatory drug; ISS, International Staging System; KRd, carfilzomib in combination with lenalidomide and dexamethasone; PI, proteasome inhibitor; Q, quartile.

^a^ Staging at the initiation of carfilzomib treatment was calculated from collected laboratory test values according to the ISS [22].

^b^ Percentage is relative to the number of patients with data available.

^c^ Frailty score was derived using an algorithm based on the sum of age score, modified Charlson Comorbidity Index score and ECOG PS [23].

^d^ Some patients received more than one previous therapy. Hence, the total numbers reported for each drug class may be smaller than the sum of the individual values of each drug within that class.

^e^ A patient was classified with disease refractory to a drug according to the International Myeloma Working Group definition if they met at least one of the three following criteria: the best response to any regimen containing the drug was either stable or progressive disease; the reason the treatment was stopped was progression in any regimen containing the drug; the date of relapse/progression was after the start date and within 60 days (inclusive) after the stop date of the drug in any regimen containing the drug.

^f^ Percentage was calculated based on the number of patients who previously received the indicated treatment.

### 3.2 Treatment history overall and by line of therapy

The median time since discontinuation of previous treatment was 10.3 months overall, 16.0 months and 2.1 months for those treated at 2L or 3L+, respectively. Overall, approximately two‐thirds of patients (63.7%) had received a haematopoietic stem cell transplant (Table 1). Nearly all patients (95.6%) had been treated with bortezomib, of whom approximately one‐third (30.6%) were refractory to it. Approximately one‐third of patients (34.2%) had received lenalidomide, and 57.3% were refractory to it. Prior exposure to lenalidomide was reported for 10.9% and 69.3% of patients receiving KRd at 2L or 3L+, respectively, of whom 32.0% and 63.2%, respectively, had disease refractory to lenalidomide. A small proportion (8.9%) of patients received daratumumab, of whom 94.1% had refractory disease. Few patients (1.0%) had previously received carfilzomib.

### 3.3 Response overall and by line of therapy

Best overall response was available for 360 patients; 95.3% of these assessments (343 of 360) were conducted by the investigator using IMWG criteria. Overall response rate (ORR) (95% CI) was 83.6% (79.4–87.3); a complete response (CR) or better and a very good partial response (VGPR) or better were achieved in 31.7% and 66.7% of patients, respectively (Table 2). Responses were better when KRd was given at earlier (ORR [95% CI]: 2L, 85.3% [79.9–89.7]; VGPR or better: 2L, 71.6%) rather than later lines (ORR [95% CI]: 3L+, 81.0% [73.6–87.1]; VGPR or better: 3L+, 59.2%).

**TABLE 2:** Best overall response to KRd overall and by line of therapy

**Response**	**2L (*n* = 230)**	**3L+ (*n* = 153)**	**Overall (*N* = 383)**
Patients with disease response assessment	218 (94.8)	142 (92.8)	360 (94.0)
ORR^a^	186 (85.3)	115 (81.0)	301 (83.6)
95% CI	79.9–89.7	73.6–87.1	79.4–87.3
Best overall response^a^			
CR or better	80 (36.7)	34 (23.9)	114 (31.7)
VGPR or better	156 (71.6)	84 (59.2)	240 (66.7)
sCR	11 (5.0)	6 (4.2)	17 (4.7)
CR	69 (31.7)	28 (19.7)	97 (26.9)
VGPR	76 (34.9)	50 (35.2)	126 (35.0)
PR	30 (13.8)	31 (21.8)	61 (16.9)
MR	3 (1.4)	3 (2.1)	6 (1.7)
SD	16 (7.3)	8 (5.6)	24 (6.7)
PD	11 (5.0)	14 (9.9)	25 (6.9)
NE	2 (0.9)	2 (1.4)	4 (1.1)

Data are presented as *n* (%).

Percentages are subject to rounding.

Abbreviations: 2L, second line; 3L+, third line or later; CI, confidence interval; CR, complete response; KRd, carfilzomib in combination with lenalidomide and dexamethasone; MR, minimal response; NE, not evaluable; ORR, overall response rate; PD, progressive disease; PR, partial response; sCR, stringent complete response; SD, stable disease; VGPR, very good partial response.

^a^ Percentage is out of patients who had a disease response assessment.

### 3.4 Carfilzomib administration and discontinuation

Both overall and by line of therapy, most patients (overall, 90.6%; 2L, 90.0%; 3L+, 91.5%) received carfilzomib in line with the European label: twice weekly at a dose of 27 mg/m^2^ (starting dose of 20 mg/m^2^ in cycle 1). Administration of carfilzomib on a once‐weekly schedule was planned in only 5.0% of patients (2L, 6.1%; 3L+, 3.3%); however, in practice, 14.1% of patients received carfilzomib once weekly (2L, 15.2%; 3L+, 12.4%). Carfilzomib was administered once every 2 weeks to 5.2% of patients (2L, 6.5%; 3L+, 3.3%).

In practice, the median (range) average dose of carfilzomib per administration across all doses administered was 26.8 (17.8–68.6) mg/m^2^ (27.0 [17.6–70.0] mg/m^2^ when excluding doses on days 1 and 2 of cycle 1) and 74.9% of patients received a relative dose intensity of 80% or greater. Proportions were similar by treatment line: 74.7% of patients at 2L and 75.2% of patients at 3L+ received a relative dose intensity of 80% or greater.

**FIGURE 1:**
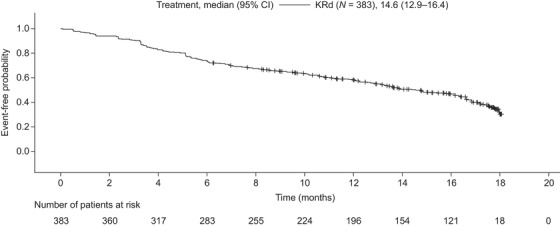
Kaplan–Meier plot of overall time to carfilzomib discontinuation for patients receiving KRd. An event is defined as the discontinuation of carfilzomib treatment. Patients who have not discontinued carfilzomib treatment are censored on their last recorded non‐zero dose date (indicated by tick marks). Abbreviations: CI, confidence interval; KRd, carfilzomib in combination with lenalidomide and dexamethasone

At a median follow‐up of 17.7 months, 224 patients (58.5%) receiving KRd had discontinued carfilzomib treatment. The median (95% CI) time to carfilzomib discontinuation was 14.6 (12.9–16.4) months overall (Figure 1), 15.9 (12.6–17.2) months for patients treated at 2L, 14.8 (8.3–16.9) months for 3L, 14.8 (12.0–17.5) months for the fourth line and 10.8 (4.4–15.8) months for fifth or later lines. The main reasons for carfilzomib discontinuation were disease progression/refractory disease (overall, 31.3%; 2L, 28.0%; 3L+, 35.4%), adverse events (AEs) (overall, 25.0%; 2L, 22.4%; 3L+, 28.3%) and achievement of the required level of treatment response (overall, 22.8%; 2L, 25.6%; 3L+, 19.2%).

### 3.5 Safety

TEAEs (≥ grade 3) occurred in 184 patients (48.0%) and treatment‐related TEAEs (≥ grade 3) occurred in 94 patients (24.5%). The most common treatment‐related TEAEs were neutropenia (6.8%), infections and infestations (4.4%) and anaemia (4.2%) (Table 3). Treatment‐related serious AEs (SAEs) occurred in 46 patients (12.0%). For 18 patients (4.7%), treatment‐related TEAEs led to carfilzomib discontinuation. Fatal AEs occurred in two patients (0.5%): one event of heart failure and one of pulmonary embolism; both patients had initiated KRd at 2L. The safety profile was similar regardless of whether KRd was administered at 2L or 3L+. More treatment‐related TEAEs occurred in patients who initiated KRd at 2L, except for neutropenia (3L+, 8.5%; 2L, 5.7%). However, a greater proportion of patients at 3L+ (*n* = 9; 5.9%) than patients at 2L (*n* = 9, 3.9%) experienced treatment‐related TEAEs leading to carfilzomib discontinuation.

**TABLE 3:** Safety data overall and by line of therapy

	**2L (*n* = 230)**	**3L+ (*n* = 153)**	**Overall (*N* = 383)**
TEAEs (≥ grade 3)	106 (46.1)	78 (51.0)	184 (48.0)
SAEs	73 (31.7)	52 (34.0)	125 (32.6)
AEs leading to discontinuation of carfilzomib	20 (8.7)	21 (13.7)	41 (10.7)
Fatal AEs	6 (2.6)	8 (5.2)	14 (3.7)
Treatment‐related TEAEs (≥ grade 3)	58 (25.2)	36 (23.5)	94 (24.5)
SAEs	30 (13.0)	16 (10.5)	46 (12.0)
AEs leading to discontinuation of carfilzomib	9 (3.9)	9 (5.9)	18 (4.7)
Fatal AEs	2 (0.9)	0 (0.0)	2 (0.5)
Most common (≥5% of any subgroup or overall by SOC) treatment‐related TEAEs (≥ grade 3), classified by HLGT or PT			
**Blood and lymphatic system disorders**	**26 (11.3)**	**18 (11.8)**	**44 (11.5)**
Anaemia^a^	11 (4.8)	5 (3.3)	16 (4.2)
Cytopenia^a^	3 (1.3)	0 (0.0)	3 (0.8)
Febrile neutropenia^a^	0 (0.0)	1 (0.7)	1 (0.3)
Leukopenia^a^	1 (0.4)	1 (0.7)	2 (0.5)
Neutropenia^a^	13 (5.7)	13 (8.5)	26 (6.8)
Thrombocytopenia^a^	10 (4.3)	3 (2.0)	13 (3.4)
**Infections and infestations**	**13 (5.7)**	**4 (2.6)**	**17 (4.4)**
Bacterial infectious disorders^b^	1 (0.4)	1 (0.7)	2 (0.5)
Infections^b^	12 (5.2)	3 (2.0)	15 (3.9)
Viral infectious disorders^b^	1 (0.4)	1 (0.7)	2 (0.5)

Data presented as *n* (%). *n* represents the number of patients who experienced one or more AEs. Patients were counted only once for each PT, HLGT or SOC level. The total number at the SOC level may be lower than the sum of the individual numbers reported at the HLGT or PT level because one patient could experience multiple events.

Percentages are subject to rounding.

AEs were coded using Medical Dictionary for Regulatory Activities version 23.0 and graded using National Cancer Institute Common Terminology Criteria for AEs version 4.03.

Abbreviations: 2L, second line; 3L+, third line or later; AE, adverse event; HLGT, High‐Level Group Term; K, carfilzomib; PT, Preferred Term; SAE, serious adverse event; SOC, System Organ Class; TEAE, treatment‐emergent adverse event.

^a^ Treatment‐related TEAE (≥ grade 3) classified by PT.

^b^ Treatment‐related TEAE (≥ grade 3) classified by HLGT.

### 3.6 Anti‐CD38 antibody‐refractory subgroup

#### 3.6.1 Patient demographics and disease characteristics

Of the 35 patients who had previously received anti‐CD38 antibodies (daratumumab or isatuximab), 33 had anti‐CD38 antibody‐refractory disease at the time of KRd initiation (Table S1). Of these, 16 and 17 patients received KRd at 2L/3L and at fourth line or later (4L+), respectively. The median age was 66.0 years (2L/3L, 59.5 years; 4L+, 69.0 years). When compared with the overall KRd population, a higher proportion of patients in this subgroup were frail (52.2% vs. 30.7%, respectively) (Table S1 and Table 1).

#### 3.6.2 Treatment history overall and by line of therapy

The median number of previous lines of therapy was higher in this subgroup than in the overall KRd population (3.0 vs. 1.0, respectively) (Table S2 and Table 1). Anti‐CD38 antibodies were mainly (84.8%) given as continuous therapy in this subgroup, of whom one‐third of patients (33.3%) received it as monotherapy (Table S2). Approximately half of the patients (48.5%) were double‐class refractory and over one‐third (39.4%) were triple‐class refractory.

#### 3.6.3 Response overall and by line of therapy

In the 27 patients who had a response assessment, the ORR (95% CI) was 66.7% (46.0–83.5), and 44.4% of individuals had a VGPR or better (Table 4). ORRs (95% CI) were higher when KRd was given at earlier (2L/3L, 75.0% [42.8–94.5]) rather than later (4L+, 60.0% [32.3–83.7]) treatment lines.

**TABLE 4:** Best overall response to KRd in the anti‐CD38 antibody‐refractory subgroup of patients

	**2L/3L (*n* = 16)**	**4L+ (*n* = 17)**	**Overall (*n* = 33)**
Patients with disease response assessment	12 (75.0)	15 (88.2)	27 (81.8)
ORR^a^	9 (75.0)	9 (60.0)	18 (66.7)
95% CI	42.8–94.5	32.3–83.7	46.0–83.5
Best overall response^a^			
CR or better	4 (33.3)	1 (6.7)	5 (18.5)
VGPR or better	8 (66.7)	4 (26.7)	12 (44.4)
sCR	2 (16.7)	0 (0.0)	2 (7.4)
CR	2 (16.7)	1 (6.7)	3 (11.1)
VGPR	4 (33.3)	3 (20.0)	7 (25.9)
PR	1 (8.3)	5 (33.3)	6 (22.2)
MR	0 (0.0)	1 (6.7)	1 (3.7)
SD	1 (8.3)	2 (13.3)	3 (11.1)
PD	2 (16.7)	2 (13.3)	4 (14.8)
NE	0 (0.0)	1 (6.7)	1 (3.7)

Data presented as *n* (%) unless stated otherwise.

Percentages are subject to rounding.

Abbreviations: 2L, second line; 3L, third line; 4L+, fourth line or later; CI, confidence interval; CR, complete response; KRd, carfilzomib in combination with lenalidomide and dexamethasone; MR, minimal response; NE, not evaluable; ORR, overall response rate; PD, progressive disease; PR, partial response; sCR, stringent complete response; SD, stable disease; VGPR, very good partial response.

^a^ Percentage is out of patients who had a disease response assessment.

#### 3.6.4 Carfilzomib use and discontinuation

The median time to carfilzomib discontinuation was 6.9 months overall. Patients who received carfilzomib at 2L/3L had a longer median time to discontinuation than those who received it at 4L+ (12.1 months vs. 4.4 months, respectively).

#### 3.6.5 Safety

TEAEs occurred in 20 patients (60.6%) in this subgroup, and treatment‐related TEAEs occurred in 11 patients (33.3%) (Table S3). Overall, the most common treatment‐related TEAEs were neutropenia (*n* = 2, 6.1%) and anaemia (*n* = 2, 6.1%). Treatment‐related SAEs were reported in six patients. No treatment‐related events were fatal. Treatment‐related TEAEs led to carfilzomib discontinuation in two patients who initiated KRd at 4L+.

### 3.7 Lenalidomide‐exposed subgroup

#### 3.7.1 Patient and disease characteristics

Of the 131 patients who had previously received lenalidomide, 75 were lenalidomide‐refractory at KRd initiation (LR subgroup), and more (53.3%) received KRd at 4L+ than at 2L/3L (46.7%) (Table S4). Median age at KRd initiation was slightly lower in the not lenalidomide‐refractory (NLR) subgroup than in the LR subgroup (62.5 vs. 66.0 years, respectively). Relatively more patients in the NLR (57.1%) subgroup than in the LR subgroup (41.4%) or the overall KRd population (49.1%) had International Staging System stage I (Table S4 and Table 1). There were relatively more fit patients in the NLR subgroup (45.2%) than in the LR subgroup (18.2%) or the overall KRd population (33.3%).

#### 3.7.2 Treatment history overall and by line of therapy

The median number of previous lines of therapy in the overall KRd population was lower (1.0) (Table 1) than in the NLR or the LR subgroups (2.0 and 3.0, respectively) (Table S5). Relatively more patients in the LR subgroup had been exposed to daratumumab (21.3%) than in the NLR subgroup (8.9%) or the overall KRd population (8.9%) (Table S5 and Table 1). Most patients (69.6%) in the NLR subgroup were not refractory to any agents, whereas 44.0% of the LR subgroup were double‐class refractory, mainly to an IMiD + PI (Table S5).

#### 3.7.3 Response overall and by line of therapy

In patients who had a response assessment, the ORR (95% CI) was 68.1% (55.8–78.8) in the LR subgroup, with 46.4% achieving a VGPR or better (Table 5); the ORR (95% CI) was higher in the NLR subgroup (82.0% [68.6–91.4]), with 64.0% achieving a VGPR or better.

**TABLE 5:** Best overall response to KRd in the lenalidomide‐exposed subgroup of patients

**Response**	**Lenalidomide exposed: not refractory**			**Lenalidomide exposed: refractory**		
	**2L/3L**	**4L+**	**Overall**	**2L/3L**	**4L+**	**Overall**
	**(*n* = 41)**	**(*n* = 15)**	**(*n* = 56)**	**(*n* = 35)**	**(*n* = 40)**	**(*n* = 75)**
Patients with a disease response assessment	38 (92.7)	12 (80.0)	50 (89.3)	30 (85.7)	39 (97.5)	69 (92.0)
ORR^a^	31 (81.6)	10 (83.3)	41 (82.0)	19 (63.3)	28 (71.8)	47 (68.1)
95% CI	65.7–92.3	51.6–97.9	68.6–91.4	43.9–80.1	55.1–85.0	55.8–78.8
Best overall response^a^						
CR or better	10 (26.3)	2 (16.7)	12 (24.0)	6 (20.0)	6 (15.4)	12 (17.4)
VGPR or better	27 (71.1)	5 (41.7)	32 (64.0)	15 (50.0)	17 (43.6)	32 (46.4)
sCR	0 (0.0)	2 (16.7)	2 (4.0)	2 (6.7)	1 (2.6)	3 (4.3)
CR	10 (26.3)	0 (0.0)	10 (20.0)	4 (13.3)	5 (12.8)	9 (13.0)
VGPR	17 (44.7)	3 (25.0)	20 (40.0)	9 (30.0)	11 (28.2)	20 (29.0)
PR	4 (10.5)	5 (41.7)	9 (18.0)	4 (13.3)	11 (28.2)	15 (21.7)
MR	1 (2.6)	1 (8.3)	2 (4.0)	1 (3.3)	1 (2.6)	2 (2.9)
SD	1 (2.6)	0 (0.0)	1 (2.0)	5 (16.7)	4 (10.3)	9 (13.0)
PD	4 (10.5)	1 (8.3)	5 (10.0)	5 (16.7)	5 (12.8)	10 (14.5)
NE	1 (2.6)	0 (0.0)	1 (2.0)	0 (0.0)	1 (2.6)	1 (1.4)

Data presented as *n* (%) unless stated otherwise.

Percentages are subject to rounding.

Abbreviations: 2L, second line; 3L, third line; 4L+, fourth line or later; CI, confidence interval; CR, complete response; KRd, carfilzomib in combination with lenalidomide and dexamethasone; MR, minimal response; NE, not evaluable; ORR, overall response rate; PD, progressive disease; PR, partial response; sCR, stringent complete response; SD, stable disease; VGPR, very good partial response.

^a^ Percentage is out of patients who had a disease response assessment.

#### 3.7.4 Carfilzomib use and discontinuation

At a median follow‐up of 17.7 months in the LR subgroup and 17.8 months in the NLR subgroup, 55 patients (73.3%) and 37 patients (66.1%), respectively, had discontinued carfilzomib. The median (95% CI) time to carfilzomib discontinuation was shorter in the LR subgroup (11.1 [5.3–13.9] months) than in the NLR subgroup (14.4 [5.8–16.9] months). The most common reason for carfilzomib discontinuation was disease progression (43.6%) and AEs (29.7%) in the LR and NLR subgroups, respectively. In patients who received KRd at 2L/3L, the median (95% CI) time to carfilzomib discontinuation was 11.1 (4.0–17.3) months in the LR subgroup and 13.1 (4.7–16.9) months in the NLR subgroup. In individuals who received KRd at 4L+, the median (95% CI) time to carfilzomib discontinuation was 10.8 (5.1–13.6) months and 15.1 (2.4–17.8) months in the LR and NLR subgroups, respectively.

#### 3.7.5 Safety

TEAEs occurred in 38 patients (50.7%) and 31 patients (55.4%) in the LR and NLR subgroups, respectively (Table S6). Treatment‐related TEAEs occurred in 17 patients (22.7%) and 16 patients (28.6%), respectively. The most common treatment‐related TEAEs in the LR and NLR subgroups, respectively, were neutropenia (8.0% and 8.9%), hypertension (8.0% and 5.4%) and infections and infestations (1.3% and 5.4%). Treatment‐related SAEs were less frequent in the LR subgroup than in the NLR subgroup (8.0% vs. 16.1%). No treatment‐related fatal events were reported in either subgroup. Three patients in each subgroup (LR, 4.0%; NLR, 5.4%) experienced a treatment‐related TEAE leading to carfilzomib discontinuation.

## 4 DISCUSSION

This real‐world study showed that the use, effectiveness and safety of KRd in patients with RRMM from 11 countries were largely similar when compared to what is observed in the clinical trial setting. ORR response was high and overall treatment responses were better when KRd was prescribed at earlier rather than later lines of therapy: more patients reported a CR or better and a VGPR or better when receiving KRd at 2L than at 3L+. The safety profile of KRd was consistent with previous reports [13, 14]. KRd use was in accordance with the EU label, maintaining treatment for long durations.

Daratumumab use is likely to increase following its approval in first‐line combination therapy, such as daratumumab–lenalidomide–dexamethasone, and with European Hematology Association and European Society for Medical Oncology guidelines recommending its use in this setting [15, 17]. Multi‐drug regimens, including KRd, are increasingly being used at first relapse rather than two‐drug regimens combining dexamethasone with either an IMiD or PI [20]. Although the small number of patients in the anti‐CD38 antibody‐refractory subgroup limits our ability to draw firm conclusions, our data suggest that KRd could be a potential treatment against anti‐CD38 antibody‐refractory disease, aligning with previously published data. A multicentre, retrospective study found that carfilzomib‐based regimens are suitable as the first subsequent line of therapy for patients with anti‐CD38 antibody‐refractory RRMM, reporting an ORR of 32%.[11] In our study, KRd resulted in an ORR of 66.7% in the anti‐CD38 antibody‐refractory subgroup. These patients achieved deeper responses when KRd was administered in earlier lines than in later lines of therapy (ORR: 2L/3L, 75.0%; 4L+, 60.0%), emphasising the importance of optimising therapy selection and sequencing in the relapsed setting.

The ASPIRE trial compared KRd with Rd in patients with RRMM who had received one to three previous treatments [14]. The ORR for patients treated with KRd in our study was in line with that found in ASPIRE (83.6% and 87.1%, respectively). Similarly, for patients with LR disease, the ORR aligns with that reported in ASPIRE (68.1% and 69.0%, respectively); these are encouraging outcomes in this difficult‐to‐treat subset of patients [21]. Although the two studies above are not directly comparable, patient and disease characteristics were somewhat similar [14]. Notably, the median number of previous lines of therapy indicates that patients initiated KRd earlier in this real‐world setting than in ASPIRE (1.0 vs. 2.0, respectively) [14].

Our data provide insight into the real‐world use of carfilzomib in patients with RRMM, including the use of dosing schedules that differ from the EU label. In this study, only a small proportion of patients (5.2%) had their carfilzomib dose adjusted from twice weekly to once every 2 weeks. Dose adjustments could be more widely considered for certain patients, especially those with comorbidities; this could mitigate the proportion of those who experience TEAEs of grade 3 and above.

Our study contributes valuable real‐world data for an important RRMM population that is underrepresented in clinical trials. Our findings may also be considered more generalisable to the real‐world population of patients with RRMM than those reported in clinical trials, owing to the large geographical scale of our study. We report data for different exposure and refractoriness subpopulations that may benefit from different combination therapies; these data may support clinicians in tailoring treatment decisions for their patients.

Reflecting an inherent limitation of real‐world studies, patient demographics and disease characteristics data were not available for a sizeable proportion of patients (approximately 40%–70%) and should be interpreted with caution. Longer follow‐ups beyond 3 years and more global data would be required to gain further insight into the use of carfilzomib in the patient population. Future studies could be conducted in patients in earlier lines following refractoriness to anti‐CD38 antibodies or lenalidomide.

In conclusion, our real‐world study further supports the use of KRd for RRMM and in populations with disease refractory to anti‐CD38 antibodies or lenalidomide. Our data demonstrate that KRd achieves greater responses in earlier rather than later lines of therapy, which emphasises the importance of optimising therapies in patients with RRMM.

## AUTHOR CONTRIBUTIONS

SW designed the research study; XL, EK, TK, ET, JC, RZ, and SB performed the research; XL, ET, JC, RZ, AB, TL and SW analysed the data; SW and TL drafted the manuscript. All authors provided a critical review.

## CONFLICT OF INTEREST

Xavier Leleu received honoraria from Amgen, BMS, CARsgen Therapeutics, Celgene, Gilead, Janssen, Karyopharm Therapeutics, Merck, Oncopeptides, Roche and Takeda. Eirini Katodritou received honoraria and research funding from Amgen and Janssen‐Cilag; held membership on boards of directors or advisory committees for Amgen and Janssen‐Cilag; received expenses and research funding from Genesis Pharma and Takeda; and received research funding from AbbVie and Karyopharm Therapeutics. Thomas Kuehr received consultancy fees and honoraria from Incyte and Janssen; received honoraria and fees to cover travel, accommodation and expenses from Bayer and Lilly; and received honoraria from AbbVie, Amgen, Celgene, Merck, Novartis, Roche and Takeda. Evangelos Terpos received grants, personal fees and non‐financial support from Amgen regarding this work; received grants, personal fees and non‐financial support from Amgen, Celgene/Genesis Pharma, Janssen and Takeda; and received personal fees from AbbVie, BMS, GSK and Novartis, outside the submitted work. Jo Caers received honoraria from Amgen, Celgene, Janssen and Takeda; held membership on boards of directors or advisory committees for Amgen, Celgene, Janssen and Takeda. Renato Zambello held membership on boards of directors or advisory committees for Celgene and Janssen. Alessandra Brescianini is an employee of Amgen and an equity holder. Tony Liang is an employee of Parexel and received funding from Amgen. Sally Wetten was an employee of Amgen and an equity holder at the time of this work. Sorina N. Badelita received consultancy fees, fees to cover travel, accommodation and expenses from Amgen, Janssen and Takeda; and consultancy fees and honoraria from Novartis.

## ETHICS STATEMENT

This study was approved by appropriate local institutional review boards/independent ethics committees when required by local regulations and was conducted in accordance with the principles of the Declaration of Helsinki and Good Clinical Practice guidelines issued by the International Council for Harmonisation. Written informed consent was obtained from all patients before enrolment.

## Supporting information

Supporting Information

## Data Availability

The data that support the findings of this study are available from the corresponding author upon reasonable request. Qualified researchers may request data from Amgen studies. Complete details are available at the following: http://www.amgen.com/datasharing.
