# Supplementary material for: Real‐world use of carfilzomib combined with lenalidomide and dexamethasone in patients with multiple myeloma in Europe and Israel
Source: EJHaem. 2022 Nov 6;4(1):174–83. doi: 10.1002/jha2.595 (PMC9928790; doi:10.1002/jha2.595)
Supplement: Supplementary file 1 — Supporting Information [file JHA2-4-174-s001.docx]

## Supplementary materials

## Table S1 Patient and disease baseline characteristics for the anti-CD38 antibody-refractory subgroup

|  | 2L/3L  (*n* = 16) | 4L+  (*n* = 17) | Overall  (*n* = 33) |
| --- | --- | --- | --- |
| Sex, male | 9 (56.3) | 10 (58.8) | 19 (57.6) |
| Age at KRd initiation, years, median (Q1–Q3) | 59.5 (52.0–71.0) | 69.0 (64.0–71.0) | 66.0 (58.0–71.0) |
| ISS stage^a^ at carfilzomib initiation | 2 (12.5) | 7 (41.2) | 9 (27.3) |
| I^b^ | 1 (50.0) | 4 (57.1) | 5 (55.6) |
| II^b^ | 1 (50.0) | 2 (28.6) | 3 (33.3) |
| III^b^ | 0 (0.0) | 1 (14.3) | 1 (11.1) |
| ECOG PS at carfilzomib initiation | 11 (68.8) | 12 (70.6) | 23 (69.7) |
| 0–1^b^ | 6 (54.5) | 8 (66.7) | 14 (60.9) |
| 2–3^b^ | 5 (45.5) | 4 (33.3) | 9 (39.1) |
| 4^b^ | 0 (0.0) | 0 (0.0) | 0 (0.0) |
| Derived frailty score^c^ | 11 (68.8) | 12 (70.6) | 23 (69.7) |
| Fit^b^ | 2 (18.2) | 2 (16.7) | 4 (17.4) |
| Intermediate^b^ | 3 (27.3) | 4 (33.3) | 7 (30.4) |
| Frail^b^ | 6 (54.5) | 6 (50.0) | 12 (52.2) |
| Cytogenetic risk at diagnosis | 13 (81.3) | 3 (17.6) | 16 (48.5) |
| High^b^ | 8 (61.5) | 1 (33.3) | 9 (56.3) |
| Standard/intermediate^b^ | 3 (23.1) | 0 (0.0) | 3 (18.8) |
| Not available^b^ | 2 (15.4) | 2 (66.7) | 4 (25.0) |

Data presented as *n* (%) unless stated otherwise.

Percentages are subject to rounding.

2L, second line; 3L, third line; 4L+, fourth line or later; ECOG PS, Eastern Cooperative Oncology Group performance status; ISS, International Staging System; KRd, carfilzomib in combination with lenalidomide and dexamethasone; Q, quartile.

^a^Staging at initiation of carfilzomib treatment was calculated from collected laboratory test values according to the ISS.^21^

^b^Percentage is relative to the number of patients with data.

^c^Frailty score was derived using an algorithm based on the sum of age score, modified Charlson Comorbidity Index score and ECOG PS.^22^

## Table S2 Treatment history for the anti-CD38 antibody-refractory subgroup

|  | 2L/3L  (*n* = 16) | 4L+  (*n* = 17) | Overall  (*n* = 33) |
| --- | --- | --- | --- |
| Number of prior lines of therapy, median (Q1–Q3) | 1.0 (1.0–2.0) | 4.0 (4.0–8.0) | 3.0 (1.0–4.0) |
| Type of previous therapy^a^ |  | | |
| PI | 16 (100.0) | 17 (100.0) | 33 (100.0) |
| Bortezomib | 14 (87.5) | 17 (100.0) | 31 (93.9) |
| Ixazomib | 4 (25.0) | 4 (23.5) | 8 (24.2) |
| Carfilzomib | 0 (0.0) | 1 (5.9) | 1 (3.0) |
| IMiD | 8 (50.0) | 17 (100.0) | 25 (75.8) |
| Lenalidomide  Pomalidomide  Thalidomide | 5 (31.3)  2 (12.5)  5 (31.3) | 15 (88.2)  14 (82.4)  11 (64.7) | 20 (60.6)  16 (48.5)  16 (48.5) |
| Monoclonal antibody | 16 (100.0) | 17 (100.0) | 33 (100.0) |
| Daratumumab | 15 (93.8) | 17 (100.0) | 32 (97.0) |
| Isatuximab  Elotuzumab | 1 (6.3)  0 (0.0) | 1 (5.9)  0 (0.0) | 2 (6.1)  0 (0.0) |
| Previous HSCT | 8 (50.0) | 14 (82.4) | 22 (66.7) |
| Anti-CD38 antibody given as maintenance therapy | 4 (25.0) | 2 (11.8) | 6 (18.2) |
| Induction regimen including an  anti-CD38 antibody | 3 (18.8) | 1 (5.9) | 4 (12.1) |
| Anti-CD38 antibody not given as maintenance therapy | 12 (75.0) | 16 (94.1) | 28 (84.8) |
| Monotherapy | 0 (0.0) | 11 (64.7) | 11 (33.3) |
| Combination therapy | 12 (75.0) | 9 (52.9) | 21 (63.6) |
| Refractory^b^ to any previous treatment line | 16 (100.0) | 17 (100.0) | 33 (100.0) |
| Single-class refractory^b^ | 3 (18.8) | 1 (5.9) | 4 (12.1) |
| Anti-CD38 antibody | 3 (18.8) | 1 (5.9) | 4 (12.1) |
| IMiD | 0 (0.0) | 0 (0.0) | 0 (0.0) |
| PI | 0 (0.0) | 0 (0.0) | 0 (0.0) |
| Double-class refractory^b^ | 10 (62.5) | 6 (35.3) | 16 (48.5) |
| Anti-CD38 antibody + PI  Anti-CD38 antibody + IMiD | 8 (50.0)  2 (12.5) | 2 (11.8)  4 (23.5) | 10 (30.3)  6 (18.2) |
| IMiD + PI | 0 (0.0) | 0 (0.0) | 0 (0.0) |
| Triple-class refractory^b^ | 3 (18.8) | 10 (58.8) | 13 (39.4) |
| Anti-CD38 antibody + IMiD + PI | 3 (18.8) | 10 (58.8) | 13 (39.4) |
| Not refractory | 0 (0.0) | 0 (0.0) | 0 (0.0) |

Data presented as *n* (%) unless stated otherwise.

Percentages are subject to rounding.

2L, second line; 3L, third line; 4L+, fourth line or later; HSCT, haematopoietic stem cell transplant; IMiD, immunomodulatory drug; PI, proteasome inhibitor; Q, quartile.

^a^Some patients received more than one previous therapy. Hence, the total numbers reported for each drug class, may be smaller than the sum of the individual values of each drug within that class.

^b^A patient was classified with disease refractory to a drug according to the International Myeloma Working Group definition if they met at least one of the three following criteria: the best response to any regimen containing the drug was either stable or progressive disease; the reason the treatment was stopped was progression in any regimen containing the drug; the date of relapse/progression was after the start date and within 60 days (inclusive) after the stop date of the drug in any regimen containing the drug.

**Table S3** Safety data for the anti-CD38 antibody-refractory subgroup

|  | 2L/3L  (*n* = 16) | 4L+  (*n* = 17) | Overall  (*n* = 33) |
| --- | --- | --- | --- |
| TEAEs (≥ grade 3) | 11 (68.8) | 9 (52.9) | 20 (60.6) |
| SAEs | 9 (56.3) | 6 (35.3) | 15 (45.5) |
| AEs leading to discontinuation of carfilzomib | 1 (6.3) | 4 (23.5) | 5 (15.2) |
| Fatal AEs | 3 (18.8) | 1 (5.9) | 4 (12.1) |
| Treatment-related TEAEs (≥ grade 3) | 6 (37.5) | 5 (29.4) | 11 (33.3) |
| SAEs | 4 (25.0) | 2 (11.8) | 6 (18.2) |
| AEs leading to discontinuation of carfilzomib | 0 (0.0) | 2 (11.8)^a^ | 2 (6.1)^a^ |
| Fatal AEs | 0 (0.0) | 0 (0.0) | 0 (0.0) |
| Most common (≥5% of any subgroup or overall and in ≥2 patients by SOC) treatment-related TEAEs (≥ grade 3), classified by HLGT or PT |  |  |  |
| **Blood and lymphatic system disorders** | **2 (12.5)** | **2 (11.8)** | **4 (12.1)** |
| Anaemia^b^ | 2 (12.5) | 0 (0.0) | 2 (6.1) |
| Neutropenia^b^ | 0 (0.0) | 2 (11.8) | 2 (6.1) |
| **Cardiac disorders** | **1 (6.3)** | **1 (5.9)** | **2 (6.1)** |
| Coronary artery disorders^c^ | 0 (0.0) | 1 (5.9) | 1 (3.0) |
| Heart failure^c^ | 1 (6.3) | 0 (0.0) | 1 (3.0) |
| **Vascular disorders** | **1 (6.3)** | **1 (5.9)** | **2 (6.1)** |
| Hypertension^b^ | 1 (6.3) | 0 (0.0) | 1 (3.0) |
| Thrombosis^c^ | 0 (0.0) | 1 (5.9) | 1 (3.0) |

Data presented as *n* (%). *n* represents the number of patients who experienced one or more AEs. Patients were counted only once for each PT, HLGT or SOC level. The total number at the SOC level may be lower than the sum of the individual numbers reported at HLGT or PT level, because one patient could experience multiple events.

Percentages are subject to rounding.

AEs were coded using Medical Dictionary for Regulatory Activities version 23.0 and graded using National Cancer Institute Common Terminology Criteria for AEs version 4.03.

2L, second line; 3L, third line; 4L+, fourth line or later; AE, adverse event; HLGT, High-Level Group Term; K, carfilzomib; PT, Preferred Term; SAE, serious adverse event; SOC, System Organ Class; TEAE, treatment-emergent adverse event.

^a^The following AEs by HLGT^c^ or PT^b^ led to K discontinuation: coronary artery disorders^c^ and decreased ejection fraction^b^.

^b^Treatment-related TEAE (≥ grade 3) classified by PT.

^c^Treatment-related TEAE (≥ grade 3) classified by HLGT.

## **Table S4** Patient and disease baseline characteristics for the lenalidomide-exposed subgroup

|  | Lenalidomide exposed: not refractory | | | Lenalidomide exposed: refractory | | |
| --- | --- | --- | --- | --- | --- | --- |
|  | 2L/3L  (*n* = 41) | 4L+  (*n* = 15) | Overall  (*n* = 56) | 2L/3L  (*n* = 35) | 4L+  (*n* = 40) | Overall  (*n* = 75) |
| Sex, male | 24 (58.5) | 11 (73.3) | 35 (62.5) | 21 (60.0) | 29 (72.5) | 50 (66.7) |
| Age at KRd initiation, years, median (Q1–Q3) | 64.0  (57.0–69.0) | 60.0  (53.0–69.0) | 62.5  (56.5–69.0) | 65.0  (58.0–71.0) | 66.0  (59.0–71.0) | 66.0  (59.0–71.0) |
| ISS stage^a^ at carfilzomib initiation | 9 (22.0) | 5 (33.3) | 14 (25.0) | 16 (45.7) | 13 (32.5) | 29 (38.7) |
| I^b^ | 5 (55.6) | 3 (60.0) | 8 (57.1) | 8 (50.0) | 4 (30.8) | 12 (41.4) |
| II^b^ | 2 (22.2) | 1 (20.0) | 3 (21.4) | 5 (31.3) | 4 (30.8) | 9 (31.0) |
| III^b^ | 2 (22.2) | 1 (20.0) | 3 (21.4) | 3 (18.8) | 5 (38.5) | 8 (27.6) |
| ECOG PS at carfilzomib initiation | 22 (53.7) | 9 (60.0) | 31 (55.4) | 24 (68.6) | 20 (50.0) | 44 (58.7) |
| 0–1^b^ | 16 (72.7) | 6 (66.7) | 22 (71.0) | 19 (79.2) | 17 (85.0) | 36 (81.8) |
| 2–3^b^ | 6 (27.3) | 2 (22.2) | 8 (25.8) | 5 (20.8) | 3 (15.0) | 8 (18.2) |
| 4^b^ | 0 (0.0) | 1 (11.1) | 1 (3.2) | 0 (0.0) | 0 (0.0) | 0 (0.0) |
| Derived frailty score^c^ | 22 (53.7) | 9 (60.0) | 31 (55.4) | 24 (68.6) | 20 (50.0) | 44 (58.7) |
| Fit^b^ | 10 (45.5) | 4 (44.4) | 14 (45.2) | 3 (12.5) | 5 (25.0) | 8 (18.2) |
| Intermediate^b^ | 6 (27.3) | 1 (11.1) | 7 (22.6) | 11 (45.8) | 6 (30.0) | 17 (38.6) |
| Frail^b^ | 6 (27.3) | 4 (44.4) | 10 (32.3) | 10 (41.7) | 9 (45.0) | 19 (43.2) |
| Cytogenetic risk at diagnosis | 22 (53.7) | 7 (46.7) | 29 (51.8) | 17 (48.6) | 14 (35.0) | 31 (41.3) |
| High | 8 (36.4) | 2 (28.6) | 10 (34.5) | 9 (52.9) | 3 (21.4) | 12 (38.7) |
| Standard/intermediate | 10 (45.5) | 2 (28.6) | 12 (41.4) | 7 (41.2) | 7 (50.0) | 14 (45.2) |
| Not available | 4 (18.2) | 3 (42.9) | 7 (24.1) | 1 (5.9) | 4 (28.6) | 5 (16.1) |

Data presented as *n* (%) unless stated otherwise.

Percentages are subject to rounding.

2L, second line; 3L, third line; 4L+, fourth line or later; ECOG PS, Eastern Cooperative Oncology Group performance status; ISS, International Staging System; KRd, carfilzomib in combination with lenalidomide and dexamethasone; Q, quartile.

^a^Staging at initiation of carfilzomib treatment was calculated from collected laboratory test values according to the ISS.^21^

^b^Percentage is relative to the number of patients with data available.

^c^Frailty score was derived using an algorithm based on the sum of age score, modified Charlson Comorbidity Index score and ECOG PS.^22^

## Table S5 Treatment history for the lenalidomide-exposed subgroup

|  | Lenalidomide exposed: not refractory | | | Lenalidomide exposed: refractory | | |
| --- | --- | --- | --- | --- | --- | --- |
|  | 2L/3L  (*n* = 41) | 4L+  (*n* = 15) | Overall  (*n* = 56) | 2L/3L  (*n* = 35) | 4L+  (*n* = 40) | Overall  (*n* = 75) |
| Number of prior lines of therapy, median (Q1–Q3) | 2.0  (1.0–2.0) | 3.0  (3.0–4.0) | 2.0  (1.0–3.0) | 2.0  (2.0–2.0) | 4.0  (3.0–5.5) | 3.0  (2.0–4.0) |
| Previous HSCT | 30 (73.2) | 14 (93.3) | 44 (78.6) | 16 (45.7) | 26 (65.0) | 42 (56.0) |
| Type of previous therapy^a^ |  | | | | | |
| PI | 38 (92.7) | 15 (100.0) | 53 (94.6) | 32 (91.4) | 40 (100.0) | 72 (96.0) |
| Bortezomib | 37 (90.2) | 14 (93.3) | 51 (91.1) | 31 (88.6) | 40 (100.0) | 71 (94.7) |
| Ixazomib | 0 (0.0) | 3 (20.0) | 3 (5.4) | 1 (2.9) | 4 (10.0) | 5 (6.7) |
| Carfilzomib | 1 (2.4) | 0 (0.0) | 1 (1.8) | 0 (0.0) | 2 (5.0) | 2 (2.7) |
| IMiD | 41 (100.0) | 15 (100.0) | 56 (100.0) | 35 (100.0) | 40 (100.0) | 75 (100.0) |
| Lenalidomide Thalidomide | 41 (100.0)  14 (34.1) | 15 (100.0)  8 (53.3) | 56 (100.0)  22 (39.3) | 35 (100.0)  11 (31.4) | 40 (100.0)  23 (57.5) | 75 (100.0)  34 (45.3) |
| Pomalidomide | 2 (4.9) | 4 (26.7) | 6 (10.7) | 2 (5.7) | 21 (52.5) | 23 (30.7) |
| Monoclonal antibody | 0 (0.0) | 5 (33.3) | 5 (8.9) | 5 (14.3) | 11 (27.5) | 16 (21.3) |
| Daratumumab | 0 (0.0) | 5 (33.3) | 5 (8.9) | 5 (14.3) | 11 (27.5) | 16 (21.3) |
| Elotuzumab | 0 (0.0) | 0 (0.0) | 0 (0.0) | 0 (0.0) | 0 (0.0) | 0 (0.0) |
| Isatuximab | 0 (0.0) | 0 (0.0) | 0 (0.0) | 0 (0.0) | 0 (0.0) | 0 (0.0) |
| Refractory to any previous treatment line^b,c^ |  |  |  |  |  |  |
| Lenalidomide Bortezomib | 0 (0.0)  8 (21.6) | 0 (0.0)  5 (35.7) | 0 (0.0)  13 (25.5) | 35 (100.0)  18 (58.1) | 40 (100.0)  20 (50.0) | 75 (100.0)  38 (53.5) |
| Daratumumab | 0 (-) | 5 (100.0) | 5 (100.0) | 5 (100.0) | 10 (90.9) | 15 (93.8) |
| Isatuximab | 0 (-) | 0 (-) | 0 (-) | 0 (-) | 0 (-) | 0 (-) |
| Single-class refractory^b^ | 5 (12.2) | 2 (13.3) | 7 (12.5) | 14 (40.0) | 17 (42.5) | 31 (41.3) |
| IMiD  PI  Anti-CD38 antibody | 1 (2.4)  4 (9.8)  0 (0.0) | 0 (0.0)  2 (13.3)  0 (0.0) | 1 (1.8)  6 (10.7)  0 (0.0) | 14 (40.0)  0 (0.0)  0 (0.0) | 17 (42.5)  0 (0.0)  0 (0.0) | 31 (41.3)  0 (0.0)  0 (0.0) |
| Double-class refractory^b^ | 4 (9.8) | 4 (26.7) | 8 (14.3) | 18 (51.4) | 15 (37.5) | 33 (44.0) |
| IMiD + PI  Anti-CD38 antibody + IMiD | 4 (9.8)  0 (0.0) | 1 (6.7)  2 (13.3) | 5 (8.9)  2 (3.6) | 16 (45.7)  2 (5.7) | 13 (32.5)  2 (5.0) | 29 (38.7)  4 (5.3) |
| Anti-CD38 antibody + PI | 0 (0.0) | 1 (6.7) | 1 (1.8) | 0 (0.0) | 0 (0.0) | 0 (0.0) |
| Triple-class refractory^b^ | 0 (0.0) | 2 (13.3) | 2 (3.6) | 3 (8.6) | 8 (20.0) | 11 (14.7) |
| Anti-CD38 antibody + IMiD + PI | 0 (0.0) | 2 (13.3) | 2 (3.6) | 3 (8.6) | 8 (20.0) | 11 (14.7) |
| Not refractory | 32 (78.0) | 7 (46.7) | 39 (69.6) | 0 (0.0) | 0 (0.0) | 0 (0.0) |

Data presented as *n* (%) unless stated otherwise.

Percentages are subject to rounding.

2L, second line; 3L, third line; 4L+, fourth line or later; HSCT, haematopoietic stem cell transplant; IMiD, immunomodulatory drug; PI, proteasome inhibitor; Q, quartile.

^a^Some patients received more than one previous therapy. Hence, the total numbers reported for each drug class, may be smaller than the sum of the individual values of each drug within that class.

^b^A patient was classified with disease refractory to a drug according to the International Myeloma Working Group definition if they met at least one of the three following criteria: the best response to any regimen containing the drug was either stable or progressive disease; the reason the treatment was stopped was progression in any regimen containing the drug; the date of relapse/progression was after the start date and within 60 days (inclusive) after the stop date of the drug in any regimen containing the drug.

^c^Percentage was calculated based on the number of patients who previously received the indicated treatment.

## Table S6 Safety data for the lenalidomide-exposed subgroup

|  | Lenalidomide exposed: not refractory | | | Lenalidomide exposed: refractory | | |
| --- | --- | --- | --- | --- | --- | --- |
|  | 2L/3L  (*n* = 41) | 4L+  (*n* = 15) | Overall  (*n* = 56) | 2L/3L  (*n* = 35) | 4L+  (*n* = 40) | Overall  (*n* = 75) |
| TEAEs (≥ grade 3) | 21 (51.2) | 10 (66.7) | 31 (55.4) | 16 (45.7) | 22 (55.0) | 38 (50.7) |
| SAEs | 12 (29.3) | 8 (53.3) | 20 (35.7) | 12 (34.3) | 18 (45.0) | 30 (40.0) |
| AEs leading to discontinuation of carfilzomib | 4 (9.8) | 3 (20.0) | 7 (12.5) | 6 (17.1) | 6 (15.0) | 12 (16.0) |
| Fatal AEs | 0 (0.0) | 2 (13.3) | 2 (3.6) | 4 (11.4) | 2 (5.0) | 6 (8.0) |
| Treatment-related TEAEs (≥ grade 3) | 14 (34.1) | 2 (13.3) | 16 (28.6) | 8 (22.9) | 9 (22.5) | 17 (22.7) |
| SAEs | 7 (17.1) | 2 (13.3) | 9 (16.1) | 2 (5.7) | 4 (10.0) | 6 (8.0) |
| AEs leading to discontinuation of carfilzomib | 2 (4.9) | 1 (6.7) | 3 (5.4) | 1 (2.9) | 2 (5.0) | 3 (4.0) |
| Fatal AEs | 0 (0.0) | 0 (0.0) | 0 (0.0) | 0 (0.0) | 0 (0.0) | 0 (0.0) |
| Most common (≥5% in any subgroup or overall and in ≥2 patients by SOC) treatment-related TEAEs (≥ grade 3) by SOC, classified by HLGT or PT |  |  |  |  |  |  |
| **Blood and lymphatic system disorders** | **6 (14.6)** | **1 (6.7)** | **7 (12.5)** | **2 (5.7)** | **6 (15.0)** | **8 (10.7)** |
| Anaemia^a^  Febrile neutropenia^a^ | 2 (4.9)  1 (2.4) | 0 (0.0)  0 (0.0) | 2 (3.6)  1 (1.8) | 0 (0.0)  0 (0.0) | 2 (5.0)  0 (0.0) | 2 (2.7)  0 (0.0) |
| Neutropenia^a^ | 4 (9.8) | 1 (6.7) | 5 (8.9) | 2 (5.7) | 4 (10.0) | 6 (8.0) |
| Thrombocytopenia^a^ | 1 (2.4) | 0 (0.0) | 1 (1.8) | 0 (0.0) | 1 (2.5) | 1 (1.3) |
| **Vascular disorders** | **3 (7.3)** | **0 (0.0)** | **3 (5.4)** | **3 (8.6)** | **3 (7.5)** | **6 (8.0)** |
| Hypertension^a^ | 3 (7.3) | 0 (0.0) | 3 (5.4) | 3 (8.6) | 3 (7.5) | 6 (8.0) |
| **Infections and infestations** | **2 (4.9)** | **1 (6.7)** | **3 (5.4)** | **1 (2.9)** | **0 (0.0)** | **1 (1.3)** |
| Bacterial infectious disorders^b^ | 0 (0.0) | 1 (6.7) | 1 (1.8) | 0 (0.0) | 0 (0.0) | 0 (0.0) |
| Infections^b^ | 2 (4.9) | 0 (0.0) | 2 (3.6) | 1 (2.9) | 0 (0.0) | 1 (1.3) |
| Viral infectious disorders^b^ | 0 (0.0) | 1 (6.7) | 1 (1.8) | 0 (0.0) | 0 (0.0) | 0 (0.0) |

Data presented as *n* (%). *n* represents the number of patients who experienced one or more adverse events. Patients were counted only once for each PT, HLGT or SOC level. The total number at the SOC level may be lower than the sum of the individual numbers reported at HLGT or PT level, because one patient could experience multiple events.

Percentages are subject to rounding.

AEs were coded using Medical Dictionary for Regulatory Activities version 23.0 and graded using National Cancer Institute Common Terminology Criteria for AEs version 4.03.

2L, second line; 3L, third line; 4L+, fourth line or later; AE, adverse event; HLGT, High-Level Group Term; K, carfilzomib; PT, Preferred Term; SAE, serious adverse event; SOC, System Organ Class; TEAE, treatment-emergent adverse event.

^a^Treatment-related TEAE (≥ grade 3) classified by PT.

^b^Treatment-related TEAE (≥ grade 3) classified by HLGT.
